# Supplementary material for: Research on urban resilience assessment and driving forces based on Production-Living-Ecological Spaces: A case study of the Three Gorges Reservoir Area in China
Source: PLoS One. 2025 Jul 1;20(7):e0326770. doi: 10.1371/journal.pone.0326770 (PMC12212500; doi:10.1371/journal.pone.0326770)
Supplement: Attachment — Submitted filename: R.Report PONE PLE Spaces 24.1.25.docx [file pone.0326770.s002.docx]

| **REVIEWER’S EVALUATION REPORT** | |
| --- | --- |
| **Manuscript Number** | PONE-D-24-55110 |
| **Full Title** | Research on County Resilience Assessment and Driving Forces based on Production-Living-Ecological Spaces: A Case study of the Three Gorges Reservoirs Area in China |
| **Article Type** | Research |
| **Title** | The Title has reflected the content of the manuscript but it needs to be modified. The author can include one caption of the word “Urban” in the titlte. The new suggested Title is that “Research on **Urban** Resilience Assessment and Driving Forces based on Production-Living-Ecological Spaces: A Case study of the Three Gorges Reservoir area in China”. |
| **Abstract** | The study objectives are not clear in the Abstrct. The first 3 lines should have to explain the objectives in the abstract. Kindly incorporate the objectives fo the study are to ………….in the Abstract. |
| **Introduction** | What are the criteria to be considerd for the “ Resilient Cities”. Introduction is very vast of three and half pages including many citations. |
| **Literature Review** | The manuscript has some pertinent literatures highlighted. |
| **Conceptual**  **Map** | The manuscript has a lot of figures but it has Conceptual Map depicted with clear meaning of the locations high altitude, mid-altitude and lowland categories of three reservoirs. . |
| **Material**  **and**  **Methods** | How Digital Elevation Model was supported the spatial data collection in these three reservoirs?  Around 11 Documents have been verified for the data collection from these three reservoirs.  Missiing data were managed technically.  On Page 9, left side No. 165 and in many places in the manuscript mentioned that this article is not correct because it is not yet published. Rather the authors can say that the manuscript because it is under reviewing process. After publications, the manuscript can be an article.  This research study has three broader focus Production Space Resiilence, Living Space Resilience and Ecological Space Resilience. Of which, the Living Space Resilence has 14 Indiacators to measure them because of the majority of the stakeholders populations are living over there need to be checked with complete more pertinent indicators. |
| **Results**  **and**  **Discussion** | ArcGIS software was used to obtain the spatial distribution of the counties in the Three Gorges Reservoirs in 2010, 2015 and 2020 with five years gap.  Table 4: Wanzhou District has the highest Comprehensive Status was identified.  Table 5: Wanzhou District has the highest Comprehensive Status was identified.  Table 6: Wanzhou District has the highest Comprehensive Status was identified.  These Tables 4,5 and 6 the values have been reflected the highest Comprehensive Status have highest Resilience level in the Districts and Counties.  On Page 22, Left side No. 334 -339 have indicated that the Low, Medium and High level of Resilience in these three reservoirs.  Table 7: Production Space driver is the top most followed by the Living Space driver and Ecological Space driver.  Figure 1 is Conceptural Map  Figure 3: Sketch Map of this research study. All the figures in the Appendices part are very clear meaning of the Comprehensive Status depicting the Three Production Space, Lliving Space and Ecological Space categories as well.  Figure 7: q values were categorized into three Spaces of which Production Space has dominant in terms of economic activities of the population followed by the Living Space has greater influence in terms of majority of the population in this space than Ecological Space. |
| **References** | Both in-text and References have the numbered Citations. There is no citation problem in the manuscript. |
| **General Comment** | The authors have to correct the word article into manuscript in the whole manuscript. Take care of the spelling mistakes and other editorial errors. |
| **Final Result** | The Article can be accepted for publication. First, however, the Authors have to carry out the corrections that the Reviewer pointed out. |
