# Supplementary material for: Research on urban resilience assessment and driving forces based on Production-Living-Ecological Spaces: A case study of the Three Gorges Reservoir Area in China
Source: PLoS One. 2025 Jul 1;20(7):e0326770. doi: 10.1371/journal.pone.0326770 (PMC12212500; doi:10.1371/journal.pone.0326770)
Supplement: Attachment — Submitted filename: Response to reviewers.docx [file pone.0326770.s004.docx]

**Response to Reviewer #1**

Dear Reviewer #1,

Thank you for taking the time to review our manuscript and providing such detailed feedback. We greatly value your comments, which will significantly help us improve the quality of the paper. Below are our responses to each of your comments, along with the corresponding revisions:

1) Abstract: “The geographical agglomeration impact is studied using the ArcGIS spatial approach.” I think you can write better here the methodology because ArcGIS is just a software.

Response: Abstract: We have revised the abstract to more clearly describe the methodology. Instead of just mentioning ArcGIS, we now specifically state the spatial analysis techniques used, such as the Jenks natural breaks method and spatial autocorrelation analysis, to more accurately demonstrate our research methods.

2) Introduction: from line 34-38, I think that citing just a policy plan of a country without discussing its (scientifically studied) implications lacks meaningful insights.

Response: Introduction: from line 35-40, We have expanded the discussion of the cited policy plans and added references to research on the effects of international policy plans in other regions to provide more meaningful insights.

3) Line 54-62, I know there are many more works about resilience studies in scientific literature. Please, report more of them by doing a more accurate literature review.

Response: line 65-75, A more comprehensive literature review has been conducted. We have added relevant studies to support our arguments and reorganized the literature review section to better reflect the current state of research and its relevance to our study.

4) Lines 71-72, the sentence is not clear. What do you mean by dimensional investigations at the geographical level?

Response: lines 84-86, We have clarified the term "geographical-level dimension investigation" (hyphen added), explaining that it refers to the study of different geographical regions.

5) Your analysis of the interactions of three spatial subsystems over time is linked to this previous study on the multidimensional spatial analyses of urban resilience over years “Casali, Y., Aydin, N. Y., & Comes, T. (2024) A data-driven approach to analyze the co-evolution of urban systems through a resilience lens: A Helsinki case study. Environment and Planning B: Urban Analytics and City Science, 51(9), 2074-2091. <https://doi.org/10.1177/23998083241235246>”

Response: We have added a citation of the study by Casali et al. (2024) and discussed its relevance to our research. We also briefly compared our methods and findings with those of Casali et al. to highlight the contributions of our study.

6) Line 139: can you add the year of the beginning and end of the Three Gorges Project? This helps to have a temporal reference.

Response: line 155-158, The specific start and completion years of the Three Gorges Project have been added to provide a clear time reference.

7) 160: “the mean filling method”. Do you take the average of what exactly (spatial mean constrained on an area or the mean of all data points?) Moreover, can you report the percentage of missing data you filled ?

Response: line 179-181, We have clarified that the mean-value filling method (hyphen added) involves calculating the spatial meaning of data points. Additionally, the percentage of missing data filled using this method has been reported.

8) 182-183: “The production area is more resilient to the effects of hazards when it has superior economic efficiency and an industrial structure that makes more sense.” Please, can you explain this sentence better? What does it mean in this sentence “that makes more sense”? Do you mean that production areas are more rich and so they can rebuild and adapt after crisis easier? Please, consider to provide evidences of this (e.g. previous studies)

Response: line 201-203, We have rephrased the sentence to clarify that "more meaningful industrial structure" refers to a more diversified and efficient industrial structure. Evidence from previous studies has also been provided to support the view that such a structure enhances resilience.

9) Section 2.3.2: please, discuss this section by using previous published studies on urban resilience. I think that you should better link your ideas with definitions of urban resilience already discussed in published scientific studies.

Response: Section 2.3.2, The discussion in this section has been expanded, with more references to studies on urban resilience. Our views are now better connected to the definitions of urban resilience discussed in published scientific research.

10) Lines 211-213: why do you select those aspects precisely? Please, write in the manuscript the rationale.

Response: line 218-223, We have detailed the reasons for choosing these specific aspects in the manuscript, explaining their relevance to the research objectives and the study area’s context.

11) Equations 1 and 2: like they are written now, they create confusion. If you want to take the positive value, there is the absolute value operator (modulus) in mathematics. Moreover, specify why you select only the positive values.

Response: Equations 1 and 2: The reasons for using only positive values are as follows:

a) Data standardization: Due to the heterogeneity of data measurements, a large volume of data, and inconsistent units, direct calculation is impossible.

b) Simplified calculations: Positive values simplify the process, avoiding complexity from negative numbers.

c) Highlighting differences: Mapping data to a 0-1 range clarifies relative differences.

d) Entropy method requirements: The entropy method requires non-negative data to reflect information dispersion.

12) 277-279: Please, avoid describing a method as “effective” but rather cite the reference to studies defining it and describe the calculation.

Response: line 299-301, We avoided describing the method as "effective" and instead cited prior studies.

13) Please, write the reasons why you selected Moran’s I.

Response: Moran’s I was used to quantify spatial autocorrelation of resilience in Three Gorges Reservoir Area counties. Key points:

a) Spatial autocorrelation: Moran’s I measures clustering patterns (e.g., high-resilience or low-resilience areas)

b) Interpretation: Values >0 indicate clustering of similar values; values <0 suggest dispersion.

c) Methodology: The Jenks natural breaks method graded resilience indices, and spatial weight matrices (based on adjacency/distance) were incorporated.

14) Line 359: TYPO

Response: The spelling error in line 381 has been corrected.

15) Tables: you can approximate the numbers with fewer decimal units for better clarity.

Response: The data in the table have (corrected verb agreement) many decimal places to convey high-precision results. This reflects meticulous calculations and ensures even subtle differences are observable.

16) When presenting and discussing the results, I think it lacks more discussion with resilience properties. You report well how results increases or dicreases, but I think that your results should link more back to the resilience theories, explaining what the results mean according to urban resilience.

Response: line557-606, We expanded the discussion of resilience attributes, linking results to resilience theory and emphasizing the significance of urban resilience outcomes.

17) Moreover, I think you relate little your results with previously published studies in resilience in the discussion and conclusion. How do your results contribute to the urban resilience literature?

Response: line655-667, We better linked our results to published resilience studies, discussing contributions to the literature and implications for future research/practice.

**Response to Reviewer #2**

Dear Reviewer #2,

Thank you for reviewing our manuscript and providing valuable feedback. Your comments are highly instructive for improving the quality of our work. Below are our detailed responses to your suggestions:

1) Title has to be slightly modified

Response: We revised the title to better reflect the research focus: "Research on County-City Resilience Assessment and Driving Forces Based on Production-Living-Ecological Spaces: A Case Study of the Three Gorges Reservoir Area in China" (hyphens added for compound terms)

2) Study Objectives must be cleared in Abstract and Introduction.

Response: The abstract has been reorganized to foreground the research purpose. We explicitly state that the study aims to explore location- and territory-related challenges in production, living, and ecological spaces and highlight its theoretical and practical contributions.

3) Since the study is space concept that focus on the location, territory in these three areas Production, Living and Ecological.

Response: As a spatial-concept study, we strengthened discussions on location and territorial issues in production-living-ecology fields throughout the manuscript.

4) All the Figures and Tables have reflected relevant meaning.

Response: All figures and tables now include clear titles and concise captions. Data were rechecked to ensure accuracy and relevance to the research theme, enhancing their role in supporting readers’ understanding.

5) No Citation Problem in the manuscript.

Response: We verified all cited literature to ensure adherence to academic standards, proper formatting, and relevance to the research context.

6) Remove the word Article because it is a manuscript under review and publication process.

Response: The term "article" was removed as suggested to align with standard manuscript terminology.

7) Take care of editorial corrections

Response: We addressed editorial issues, including awkward phrasing, grammatical errors, and punctuation inconsistencies, to improve clarity, readability, and professionalism.

Thank you again for your constructive feedback. We believe these revisions significantly enhance the manuscript and welcome further suggestions to advance the publication process.
