# Supplementary material for: Research on urban resilience assessment and driving forces based on Production-Living-Ecological Spaces: A case study of the Three Gorges Reservoir Area in China
Source: PLoS One. 2025 Jul 1;20(7):e0326770. doi: 10.1371/journal.pone.0326770 (PMC12212500; doi:10.1371/journal.pone.0326770)
Supplement: Attachment — Submitted filename: Response_to_Reviewers_auresp_2.docx [file pone.0326770.s005.docx]

Dear academic editor and reviewer,

Thank you for reviewing our manuscript and providing valuable feedback. Your comments are highly instructive for improving the quality of our work. Below are our responses to each of your comments, along with the corresponding revisions:

# Response to the academic editor

## 1) Carefully revise the text of the manuscript to avoid any repeated terms and words.

Response:

We have carefully scrutinized the entire manuscript, deleted unnecessarily repetitive terms and words and retained traces of changes in the 'Revised Manuscript with Track Changes' file.

## 2) Please also make sure to provide more insights and discuss the real-life implications of your study.

Response:

In section 5.3 Contributions, we add the real-life implications of this study, such as the usefulness of the resilience evaluation system established in this study for urban risk management and how the evaluation indicators can help to improve the resilience of the supply chain of specialty agricultural products, and so on.

# Response to the Reviewer #1

## The manuscript improved from the previous version because you wrote more details on methods and discussion to resilience studies. I suggest a minor revision because I noticed you often repeat "resilience" in the same paragraph (see for example the Contribution section). Please, check once more the whole text and avoid repetitions. Instead of using "resilience", I suggest to give more insights about it. You can describe, for example, some dynamics and processes.

Response:

We have rechecked the entire manuscript and deleted the repetition of "resilience" to avoid unnecessary repetition of terminology. In addition, we have rewritten section 5.3 Contributions and added dynamic process descriptions and real-life representations of resilience, including the value of resilience metrics, urban risk management, and resilience-enhancing policymaking, among others. All traces of the changes are kept in the document 'Revised Manuscript with Track Changes'.
